# A novel tankyrase inhibitor, MSC2504877, enhances the effects of clinical CDK4/6 inhibitors

**DOI:** 10.1038/s41598-018-36447-4

**Published:** 2019-01-17

**Authors:** Malini Menon, Richard Elliott, Leandra Bowers, Nicolae Balan, Rumana Rafiq, Sara Costa-Cabral, Felix Munkonge, Ines Trinidade, Roderick Porter, Andrew D. Campbell, Emma R. Johnson, Christina Esdar, Hans-Peter Buchstaller, Birgitta Leuthner, Felix Rohdich, Richard Schneider, Owen Sansom, Dirk Wienke, Alan Ashworth, Christopher J. Lord

**Affiliations:** 1 CRUK Gene Function Laboratory and Breast Cancer Now Toby Robins Breast Cancer Research Centre, The Institute of Cancer Research, London, SW3 6JB UK; 2 The Wellcome Trust, Euston Road, London, NW1 2BE UK; 3 CRUK Beatson Institute, Switchback Rd, Bearsden, Glasgow G61 1BD UK; 4 Merck KGaA, Biopharma Research & Development, Frankfurter Str. 250, 64293 Darmstadt, Germany; 5 Present Address: UCSF Helen Diller Family Comprehensive Cancer Centre, San Francisco, 94158 USA

## Abstract

Inhibition of the PARP superfamily tankyrase enzymes suppresses Wnt/β-catenin signalling in tumour cells. Here, we describe here a novel, drug-like small molecule inhibitor of tankyrase MSC2504877 that inhibits the growth of *APC* mutant colorectal tumour cells. Parallel siRNA and drug sensitivity screens showed that the clinical CDK4/6 inhibitor palbociclib, causes enhanced sensitivity to MSC2504877. This tankyrase inhibitor-CDK4/6 inhibitor combinatorial effect is not limited to palbociclib and MSC2504877 and is elicited with other CDK4/6 inhibitors and toolbox tankyrase inhibitors. The addition of MSC2504877 to palbociclib enhances G_1_ cell cycle arrest and cellular senescence in tumour cells. MSC2504877 exposure suppresses the upregulation of Cyclin D2 and Cyclin E2 caused by palbociclib and enhances the suppression of phospho-Rb, providing a mechanistic explanation for these effects. The combination of MSC2504877 and palbociclib was also effective in suppressing the cellular hyperproliferative phenotype seen in Apc defective intestinal stem cells *in vivo*. However, the presence of an oncogenic *Kras* p.G12D mutation in mice reversed the effects of the MSC2504877/palbociclib combination, suggesting one molecular route that could lead to drug resistance.

## Introduction

Poly(ADP-ribose) polymerases (PARP) family enzymes use β-NAD^+^ to catalyze the synthesis of poly(ADP-ribose) chains on target proteins as a form of post-translational modification, known as PARylation^1^. PARP enzymes regulate a wide range of cellular functions, including roles for PARP1 and PARP2 in DNA repair and roles for PARP5A and PARP5B, also known as Tankyrase 1 and Tankyrase 2 (TNKS1,2, collectively termed tankyrases), in telomere maintenance, the control of mitosis and the regulation of Wnt signaling^1^. Exploiting these roles in the development of novel therapeutic approaches to cancer has thus far largely been driven through the discovery and clinical development of small molecule PARP1 and PARP2 inhibitors, which have recently been approved for the treatment of *BRCA1* or *BRCA2* mutant ovarian and breast cancers^2^. In addition, the demonstration that experimental “toolbox” tankyrase inhibitors can inhibit oncogenic Wnt signaling in colorectal tumour cells^3^ has driven the discovery of additional, drug-like, tankyrase inhibitors that could be used to target tumours that have constitutively active Wnt signaling, such as those with premature truncating mutations in the APC tumour suppressor protein^4^.

Tankyrases regulate canonical Wnt signaling via PARylation of AXIN, a critical member of a multicomponent protein complex including APC, that controls the concentration of β-catenin, a key mediator of Wnt signaling. The tankyrase dependent PARylation of AXIN1 causes AXIN ubiquitination via RNF146, and its eventual proteosomal degradation. This reduction in AXIN concentration impairs the activity of the β-catenin destruction complex and thus enhances Wnt signaling^3^. Consistent with this role for tankyrases in Wnt signaling regulation, small molecule inhibitors of tankyrase which impair PARylation activity by competing with β-NAD^+^ for tankyrase binding, reduce AXIN PARylation, stabilise the β-catenin destruction complex and thus inhibit Wnt signaling, even in tumour cells with *APC* mutations that otherwise have constitutive Wnt activity^3^. As well as controlling Wnt signaling, tankyrases have also been implicated in the control of Hippo signaling by modulating YAP^5^ an oncoprotein over-expressed in many cancers, which when activated binds to transcription factors including p73^6^ and Runx2^7^.

The significant potential of being able to target a relatively common oncogenic process such as Wnt signalling has led to considerable efforts to discover small molecule inhibitors that target tankyrase. These include XAV939^3^, IWR-1 and IWR-2^8^, JW74^9^, JW55^10^, WIKI4^11^, K-756^12^, the ICR series^13^, NVP-TNKS656^14^ and G007-LK^15^. Each of these inhibitors have been shown to impair Wnt signalling *in vitro*, and in some cases, elicit anti-tumour efficacy in xenograft and/or genetically engineered mouse models of cancer^15^. For example, NVP-TNKS656, a modification of XAV939, impairs the growth of Wnt-dependent lesions in the MMTV-Wnt1 mouse cancer model^14,16^, JW74 reduces tumour burden in the Apc^Min^ mouse tumour model^9^ and JW55 impairs the proliferation of intestinal stem cells with *Apc* mutations in mice^10^. Likewise, G007-LK (a JW74 derivative), impairs *in situ* colorectal tumours in mice as well as *APC* mutant human tumour cell xenografts transplanted into recipient animals^15^. However, in most cases, when used as single agents (i.e. not in combination regimens), even when at relatively high-concentrations, tankyrase inhibitors appear to only partially impair tumour growth. Furthermore, the elevated doses of tankyrase inhibitors required to elicit tumour inhibition often result in intestinal toxicity, weight loss and death in rodents^15,17^. This suggests that the use of tankyrase inhibitors in appropriate combination treatment regimens might be more appropriate as these might allow reduced doses of tankyrase inhibitors to elicit anti-tumour responses or even enhance the anti-tumour effects of additional agents. For example, *in vitro* studies have demonstrated that tankyrase inhibitors can potentiate colorectal tumour cell responses to PI3-Kinase/AKT pathway inhibitors^18^ or MAP-kinase pathway (MEK) inhibitors^19^, suggesting that additional combination approaches involving tankyrase inhibitors might be of some value.

Here, we describe the characterisation of a novel small molecule selective tankyrase inhibitor, MSC2504877. This selective agent effectively suppresses Wnt signaling, but like previously identified tankyrase inhibitors, has limited effectiveness when used as a single agent. In order to understand how MSC2504877 might be used in drug combinations, we used parallel *in vitro* RNA interference and drug sensitivity chemosensitisation screens to identify how the tumour cell response to MSC2504877 might be enhanced. This approach identified multiple different components of the canonical Wnt pathway that modulate the response to single agent tankyrase inhibitor exposure as well as demonstrating that MSC2504877 can enhance tumour cell responses to inhibitors of the G_1_ restriction point kinases CDK4 and CDK6, including the licensed cancer drug palbociclib.

## Materials and Methods

### Cell lines and cell viability assays

Cell lines were purchased from ATCC, with the exception of MCF10A *APC* mutant isogenic cells (Sigma), and were cultured according to the supplier’s instructions. STR typing of 10 loci was performed on each cell line using the GenePrint 10 system (Promega) and used to confirm the identity of cell lines prior to storage. For short term cell survival assays, 500 cells were plated in media containing 0.5% (v/v) fetal calf serum in each well of 384 well plates. 24 hrs later, media was changed to media containing small molecule inhibitors. Cells were then continuously cultured in the presence of small molecule inhibitors for a subsequent five days, at which point cell viability was estimated by the use of CellTiter-Glo® Luminescent Cell Viability reagent (Promega, used according to the Manufacturer’s instructions). For 14-day drug exposure assays, cells were plated in six well plates (500 cells per well). 24 hrs later, media was changed to media containing small molecule inhibitors; this media was changed every three days, replenishing small molecule inhibitor each time. Cells were continuously cultured for 14 days at which point cells were lysed by adding a covering volume of CellTiter-Glo® Luminescent Cell Viability reagent (CTG). CTG was then transferred to white 96 well plates for luminescent measurements as per the Manufacturer’s instructions. CTG readings were normalised to values derived from cells exposed to the drug vehicle, DMSO, generating Surviving Fractions (SFs). SF were used to generate four parameter dose response curves in Prism (Graphpad).

### Biochemical assessment of tankyrase activity by ELISA (Autoparylation assay)

For analysis of TNKS1 and 2 autoparylation and its inhibition by small molecule inhibitors, an ELISA assay was used. These were performed in 384 well Glutathione coated microtiter plates (Express capture Glutathione coated plate, Biocat, Heidelberg, Germany). Plates were pre-equilibrated with PBS and then incubated with 50 µl of 20 ng/well GST-tagged Tnks-1 (1023–1327 aa, prepared in-house) or GST-tagged Tnks-2 (873–1166 aa, prepared in-house) which was resuspended in assay buffer (50 mM HEPES, 4 mM Mg-chloride, 0.05% (v/v) Pluronic F-68, 2 mM DTT, pH 7.7). This incubation was performed overnight at 4 °C. Plates were then incubated at room temperature for 20 minutes with 50 µl blocking buffer: 0.05% (v/v) Tween-20, 0.5% (w/v) BSA in PBS. Plates were then washed three times with PBS-Tween-20. The PARylation reaction was performed in the presence/absence of tankyrase inhibitors in 50 µl reaction solution: 50 mM HEPES, 4 mM Mg-chloride, 0.05% (v/v) Pluronic F-68, 1.4 mM DTT, 0.5% (v/v) DMSO, pH 7.7 + 10 µM bio-NAD (Biolog, Life science Inst., Bremen, Germany) for 1 hour at 30 °C. The reaction was stopped by washing plates three times with PBS-Tween-20. For PAR detection, plates were incubated for 30 minutes at room temperature, with 50 µl per well of 20 ng/µl Streptavidin, HRP conjugate (MoBiTec, Göttingen, Germany) resuspended in PBS + 0.05% (v/v) Tween-20 + 0.01% (w/v) BSA. After washing three times with PBS-Tween-20, 50 µl of SuperSignal ELISA Femto Maximum sensitivity substrate solution (ThermoFisherScientific (Pierce), Bonn, Germany) was added to each well. Following a one-minute incubation at room temperature, luminescence signals (700 nm) were measured with an Envision multimode reader (Perkin Elmer LAS Germany GmbH). The full value used was the inhibitor-free reaction. The pharmacological zero value used was XAV-939 (Tocris) used at a final concentration of 5 μM. The inhibitory values (IC_50_) were determined using either the program Symyx Assay Explorer® or Condosseo® from GeneData.

### TNKS, AXIN2 ELISA

Luminex-based bead assays were employed to assess the level of TNKS and AXIN2 proteins in cell and *ex vivo* xenograft lysates. For detection of TNKS, a RIPA cell lysis buffer (10 mM Tris HCl, 150 mM sodium chloride, 1% NP40, 1% Triton X-100, 0,4% Na-Deoxycholat, 2 mM EDTA, 0,3% SDS) was used and TNKS protein was isolated from lysates by incubation with a monoclonal anti-TNKS antibody (Thermo Scientific, MA1-41011) bound to fluorescent carboxybeads. Detection of TNKS was achieved with a polyclonal anti-TNKS antibody (Santa Cruz, sc-8337) and an appropriate PE-fluorescent secondary antibody. An NP40 cell lysis buffer (20 mM Tris/HCl pH 8.0, 150 mM NaCl, 1% (v/v) NP40, 10% (v/v) Glycerol) was used for detection of AXIN2. A monoclonal AXIN2 antibody (R&D Systems #MAB6078) bound to fluorescent carboxybeads was used for isolation of AXIN2 from lysates. A polyclonal anti-AXIN2 antibody (Cell Signaling #2151) and an appropriate PE-fluorescent secondary antibody was used for detection. The amount of isolated TNKS and AXIN2 proteins were determined with a Luminex^200^ device (Luminex Corporation) according to the manufacturer’s instruction by counting 100 events per sample.

### High-throughput siRNA and small molecule inhibitor screening

To optimise experimental conditions for siRNA screening, we assessed the effectiveness of different transfection reagents and selected conditions that met the following criteria: (i) compared to a mock control (no lipid or siRNA), the transfection of non-silencing negative control siRNA caused no more than 20% cell inhibition; (ii) compared to non-silencing negative control siRNA, the transfection of PLK1–targeting siRNA caused more than 80% cell inhibition; (iii) cell confluency reached 70% within the range of 4–7 days. The later criteria allowed assays to be terminated whilst cells were in growth phase. Once optimal conditions had been established, COLO320DM cells were transfected with a Dharmacon SMARTpool 384-well plate-arrayed siRNA library designed to target 714 kinases and kinase-related genes, 320 Wnt pathway–associated genes, 80 tumor suppressor genes, and 480 genes recurrently altered in human cancers, as described in^20^. Positive control (siPLK1) and multiple negative controls (siCON1 and siCON2; Dharmacon, catalog numbers D-001210-01-20 and D-001206-14-20, and AllStar; QIAGEN, catalog number 1027281) were included on every plate. Twenty-four hours after transfection, cell culture media containing MSC2504877 (final concentration 0.5 μM) or the drug vehicle was added to cells. Cells were then cultured for five additional days, at which point cell viability was estimated using CellTiter-Glo assay (Promega). Luminescence values were processed using the cellHTS2 R package^21^. To evaluate the effect of each siRNA pool on cell viability, luminescence measurements from cells that were exposed to the drug vehicle DMSO were log2 transformed, and then centred to the median value for each plate. Plate-centred data was then scaled to the median absolute deviation (MAD) and median of the library as a whole to produce robust Z-scores. To estimate the effect of each siRNA on MSC2504877 sensitisation, luminescence measurements from cells that were exposed to the MSC2504877were log2 transformed, and then centred to the median value for each plate. Plate centered data for cells exposed to MSC2504877 was then normalised according to plate centered data for cells exposed to DMSO, generating a Drug Sensitisation score for each siRNA. DE values were then scaled to the median absolute deviation (MAD) and median of the library as a whole to produce robust Z-scores, as described previously^22^. All screens were performed in triplicate. Screens judged to have poor dynamic range (Z’ factor ≤ 0)^23^ or poorly correlated replicates (r ≤ 0.7) were excluded during an evaluation of screen quality.

For small molecule screens, cells were plated in 384 well-plates as above. Twenty-four hours later, cell culture media containing the small molecule inhibitor library plus MSC2504877 or the drug vehicle, DMSO, was added to cells. Each small molecule inhibitor was present at eight different concentrations (0.5, 1, 5, 10, 50, 100, 500 and 1000 nM). Cells were then cultured for five additional days, at which point cell viability was estimated using CellTiter-Glo assay (Promega). Luminescence values were processed as described above to produce Drug Sensitisation Z scores for each small molecule inhibitor concentration included in the drug library.

### Drug synergy estimation

MacSynergyII^24^ was used to estimate synergy volumes, using data from five day drug exposure experiments, as described above.

### Western blotting

Whole cell protein lysates were generated by lysis with NP250 buffer (20 mM Tris pH 7.6, 1 mM EDTA, 0.5% NP40, 235 mM NaCl). Following a 20-minute incubation at 4 °C, the lysates were centrifuged and the supernatants were collected. Lysates were electrophoresed using Novex precast Bis-Tris gels (Invitrogen) and Western blotted on nitrocellulose membranes. Membranes were immunoblotted at 4 °C overnight using an anti-Rb1 antibody (NEB #9309) diluted 1:1000 (v/v). After washing, membranes were then immunoblotted for 1 hour at room temperature with a secondary antibody (Li-COR, 926–32210) diluted 1:10,000 (v/v) in 5% (w/v) milk. Protein bands were visualised and quantified using the Odyssey FC imaging system and ImageStudio software (Li-COR).

### Genetically engineered mice

All *in vivo* experiments in genetically engineered mice were performed in accordance with UK Home Office guidelines (Licence 70/8646), and are subject to ethical review by the University of Glasgow. The alleles used in this study were *Villin-Cre*^*ERT2*^ ^25^, *Apc*^*fl*^ ^26^ and *Kras*^*LSL-G12D*^ ^27^. In the context of *Villin-Cre*^*ERT2*^; *Apc*^*fl/fl*^ experiments, recombination was induced through a single intraperitoneal injection of tamoxifen at 80 mgkg^−1^ on two consecutive days, while in *Villin-Cre*^*ERT2*^; *Apc*^*fl/fl*^; *Kras*^*LSL-G12D/*+^ experiments, recombination was induced with intraperitoneal injection of 80 mgkg^−1^ tamoxifen on a single occasion. In both experimental settings, pharmacological intervention began 24 hours post initial induction, with palbociclib formulated in a solution of sodium lactate (pH 4) in H_2_O, and MSC2504877 formulated in a solution of 0.5% methycellulose in H_2_O, with each respectively administered by oral gavage once or twice daily at the appropriate dose. For analysis of cellular proliferation, 250 ul of 5-bromo-2′-deoxyuridine (BrdU) (Amersham) was administered by intraperitoneal injection at 2 hrs prior to sampling.

### Immunohistochemistry/RNAscope

Immunohistochemistry or RNA *in situ* hybridisation was performed throughout this study using formalin fixed paraffin embedded tissue specimens and standard protocols. Primary antibodies used for immunohistochemistry in relation to this study were α-BrdU (BD Biosciences #347580, 1:200) and α-p21^CIP/WAF^ (HUGO-291, CNIO, Madrid). RNA *in situ* hybridisation (RNAscope, Advanced Cell Diagnostics Ltd.) was used to detect expression of Lgr5 mRNA in tissue specimens using the RNAscope 2.5 LS detection reagent according to manufacturers’ instructions.

### Xenograft tumour model

For the *in vivo* PD study, animal procedures were performed in accordance with the German Laws for Animal Welfare and reviewed and approved by the Hessian Government Body (Regierungspräsidium) in Darmstadt (Licence DA\395). Animals were acclimatised a week prior use and general health status was monitored during the experiments. Female immunodeficient CB17 SCID mice (Charles River) 5- to 7-week-old received subcutaneously implanted tumour cell suspensions of Colo320 (5 Mio Cells in 100 µl PBS + 1:1 (v/v) matrigel/animal) in the flank. Tumours were growing until a tumour volume range from 300–800 mm³ (calculated L × W × W/2 from digital calliper measurements). Mice with appropriate tumour volume were stratified into control & treatment groups (n = 5/group). Administrations of vehicle or compound (formulated in 0.5% Methocel/0.25% Tween20/water) were orally given by gavage and tumours were collected at the respective time points after treatments for subsequent analysis.

## Results

### MSC2504877 selectively inhibits tankyrase activity and growth of APC deficient cell lines

**Figure 1:**
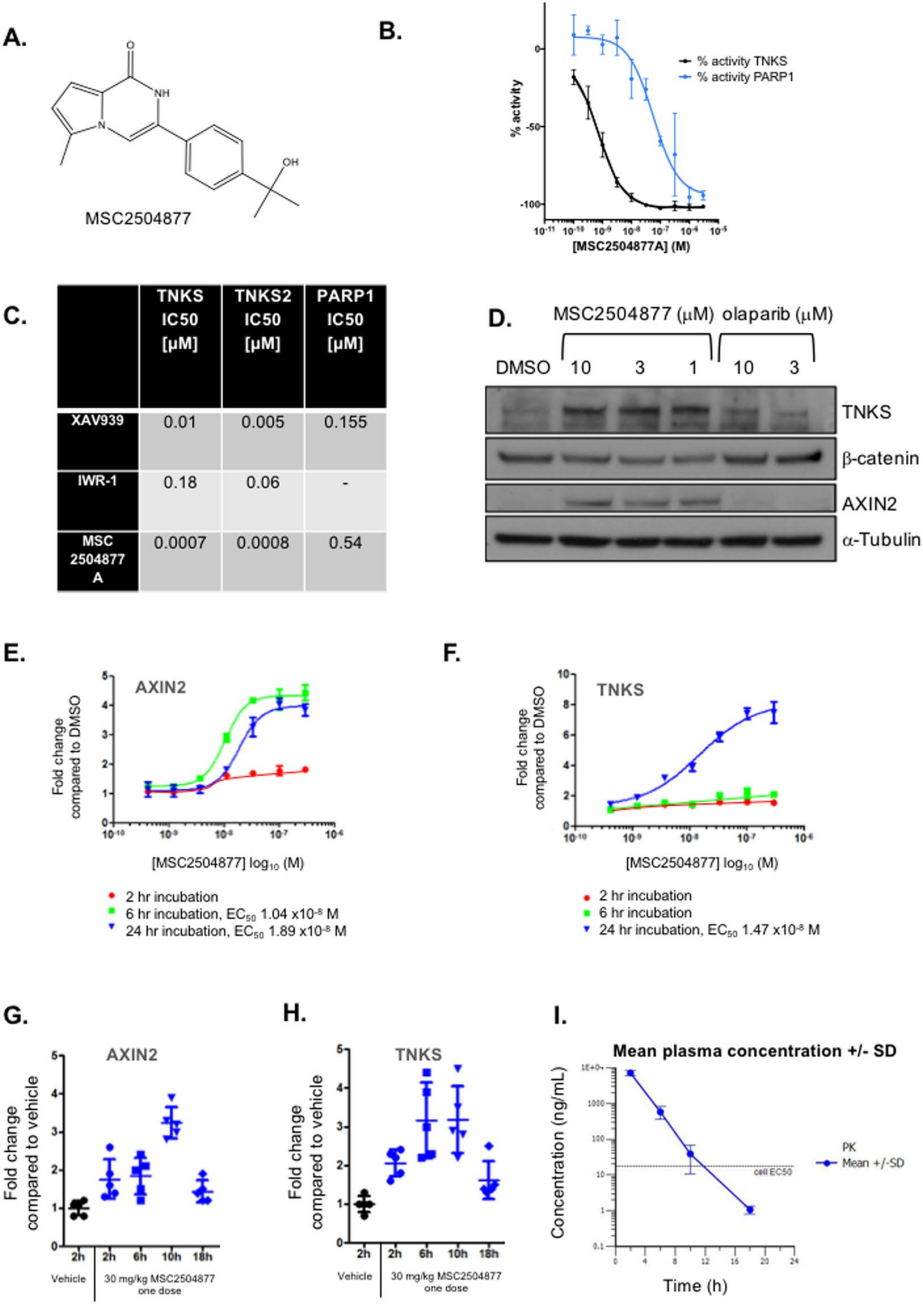
MSC2504877 is a novel, drug-like, small molecule tankyrase inhibitor. (**A**) Structure of MSC2504877. (**B**) Dose-response curve illustrating the inhibition of recombinant tankyrase (TNKS) or PARP1 with MSC2504877A. Tankyrase activity was assayed using the PARP domain of recombinant human Tankyrase (TNKS) or PARP1 in an ELISA assay. Mean dose response data from three independent experiments is shown. Error bars represent standard error of the mean (SEM). (**C**) Table illustrating IC_50_ concentrations obtained for MSC2504877A and two toolbox tankyrase inhibitors. TNKS, TNKS2 and PARP1 activity was determined as in (**B**). (**D**) Western blot illustrating tankyrase stabilisation, suppression of β-catenin and stabilisation of Axin 2 protein levels in COLO320DM (APC mutant) colorectal tumour cells exposed to MSC2504877 for 24 hours as shown. α-Tubulin was used as loading control (**E**,**F**). Luminex antibody-based detection of AXIN2 and TNKS in COLO320DM cells exposed MSC2504877 *in vitro*. Dose response data from three independent experiments are shown; error bars represent standard deviations (**G**,**H**) Luminex detection of AXIN2 and TNKS in COLO320DM xenografts. CB17 SCID mice received 30 mg/kg MSC2504877 via an oral route. At the time points indicated, mice were sacrificed and xenografts recovered. Each data point indicates data from one animal. (**I**) Plasma concentration time profile of MSC2504877 after one single oral dose of 30 mg/kg.

We identified precursors of MSC2504877 (Fig. 1A) from a small molecule screen of roughly half a million compounds, where catalytic inhibition of recombinant TNKS1 was assessed in a cell free assay (Buchstaller *et al*., manuscript in preparation). Hit-to-lead and lead optimisation efforts focused on improving the metabolic stability and solubility of the original hit matter and resulted in the identification of MSC2504877 (Buchstaller *et al*., manuscript in preparation).

Using *in vitro* biochemical assays, we found that MSC2504877 inhibited TNKS1 activity with an IC_50_ of 0.0007 µM and PARP1 activity with an IC_50_ of 0.54 µM, a 771-fold selectivity for TNKS1 (Fig. 1B,C). Inhibition of tankyrase PARylation causes a decreased level of AXIN degradation, increased activity of the β-catenin destruction complex and a decreased level of β-catenin^3^. Western blotting of lysates derived from *APC* mutant COLO320DM colorectal tumour cells exposed to MSC2504877 indicated that MSC2504877 increased AXIN2 protein levels and decreased β-catenin levels (Fig. 1D), suppressed canonical Wnt signalling in SW480 cell line that stably expresses luciferase driven by a TCF-dependent promoter (Supplementary Fig. 1) and suppressed mRNA levels of two key Wnt-driven transcripts, *RUNX2* and *AXIN2*, as assessed by qRT-PCR (Supplementary Fig. 2). MSC2504877 also caused tankyrase levels to rise (Fig. 1D), consistent with MSC2504877 inhibiting autoPARylation and subsequent degradation of tankyrase^3^. These qualitative estimates of MSC2504877 cellular activity were confirmed by the use of quantitative, antibody-based detection of AXIN2 and TNKS (using a luminex-based detection system). This suggested that MSC2504877 increased both AXIN2 (Fig. 1E) and TNKS (Fig. 1F) protein levels. We also assessed whether MSC2504877 modulated tumoral Wnt signalling *in vivo*. To do this, we administered MSC2504877 (30 mg/kg) to mice bearing *APC* mutant COLO320DM tumour cell xenografts and recovered tumours for analysis 2–18 hours later. Using the luminex-based system, we found that MSC2504877 elicited an increase in both TNKS and AXIN2 levels in tumours, peaking at 6–10 hours after drug administration and falling 18 hours after MSC2504877 treatment (Fig. 1G,H), a time course which was shifted when compared to the compound concentration time profile in mice (ttmax = 0.5 h, Cmax = 7 µg/mL; Cl = 2.72 L/h/kg, Fig. 1I), and thus in agreement with a turnover PK/PD model for a single dose administration of MSC2504877. Taken together, these observations suggested that MSC2504877 inhibited TNKS and Wnt signalling, both *in vitro* and *in vivo*.

**Figure 2:**
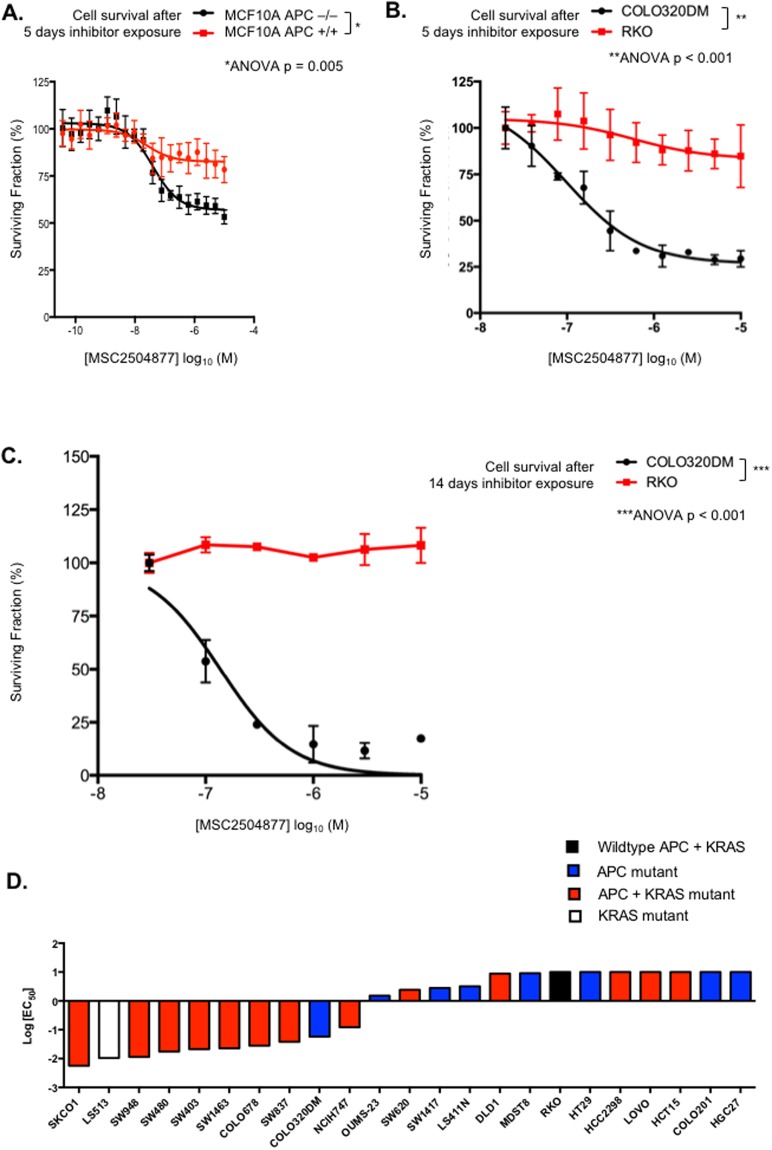
MSC2504877 elicits APC synthetic lethality. (**A**–**C**) MSC2504877 selectively targets APC defective cells. Dose response survival curves are shown for (A) MCF10A *APC* ^+/+^ and *APC* ^−/−^ isogenic cells, or (**B**) COLO320DM (APC defective) and RKO (APC wild type) colorectal tumour cells continuously exposed to MSC2504877 for a total of five days and (**C**) COLO320DM and RKO cells continuously exposed to MSC2504877 for a total of 14 days. Experiments were conducted in in media containing 0.5% (v/v) FCS. Dose response data from three independent experiments are shown; error bars represent SEM. *p* values were calculated using ANOVA. (**D**) MSC2504877 sensitivity in 23 colorectal tumour cell lines. Tumour cell lines were profiled for sensitivity to five-day exposure to MSC2504877, as in (**B**) in media containing 0.5% (v/v) FCS. Bar chart illustrating EC_50_ values is shown.

Previously described tankyrase inhibitors elicit synthetic lethality in tumour cells with *APC* tumour suppressor gene defects^3^. In the first instance, we assessed the cell inhibitory properties of MSC2504877 in an isogenic pair of *APC* wild type (*APC*^*WT*^) and APC defective (*APC*^−/−^) cells lines derived from the MCF10A non-tumour epithelial cell line. In this model, *APC* gene defects were generated by deletion of 17 bp within exon 16 of APC using gene targeting. In cell viability assays where *APC*^*WT*^ and *APC*^−/−^ isogenic cells were exposed to MSC2504877 for a five-day period, MSC2504877 significantly inhibited the survival of *APC*^−/−^ cells, compared to *APC*^*WT*^ cells (Fig. 2A, ANOVA *p* = 0.005). To assess whether this selectivity could also be achieved in colorectal tumour cell lines with naturally occurring *APC* gene mutations, we assessed the effect of MSC2504877 in *APC* mutant (p.S811* homozygous) COLO320DM and *APC* wild type RKO colorectal tumour cell lines. In both five-day and two-week drug exposure survival assays, we found that MSC2504877 had a significantly greater effect in COLO320DM cells than in RKO cells (Fig. 2B ANOVA p = 0.012 (five-day assay) and Fig. 2C ANOVA p = 0.001 (14-day assay)). We also noted that the cell inhibitory effect of MSC2504877 on COLO320DM was significantly more profound when an extended drug exposure of 14 days was used, compared to a five-day exposure (Fig. 2B,C).

Given the interest in using tankyrase inhibitors to target APC mutant colorectal cancers (CRC), we also assessed MSC2504877 sensitivity in a panel of CRC tumour cell lines. We carried out MSC2504877 sensitivity assays in 24 *APC* mutant models as well as five *APC* wild type models and found that the responses in the *APC* mutant models were varied, with some *APC* mutant models, such as COLO201, HCT15, HGC27 and LOVO, being profoundly resistant to single agent tankyrase inhibitor exposure (Fig. 2D). Although the use of isogenic cell lines clearly identified APC synthetic lethality with MSC2504877 (Fig. 2A), the varied responses in the panel of CRC tumour cell lines suggested that *APC* mutation alone might not be a fully penetrant biomarker of single agent tankyrase inhibitor response; we also did not find that the position of truncating mutations in *APC* correlated with MSC2504877, as has been suggested for other tankyrase inhibitors^4^. We assessed whether other cancer driver gene alterations found in CRC, such as those in *TP53, SMAD4, PIK3CA, KRAS, ARID1A, SOX9* and *FAM123B* correlated with sensitivity to MSC2504877, but did not find, in this tumour cell line panel at least, that gene mutations/copy number alterations in any of these strongly correlated with tankyrase inhibitor response.

### Integrated RNA interference and small molecule chemosensitisation screens identify CDK4/6 inhibition as a determinant of MSC2504877 sensitivity

To better understand what could drive sensitivity to MSC2504877, we carried out parallel RNA interference (RNAi) and small molecule/drug high-throughput chemosensitisation screens using MSC2504877 in APC mutant colorectal cancer tumour cell lines.

**Figure 3:**
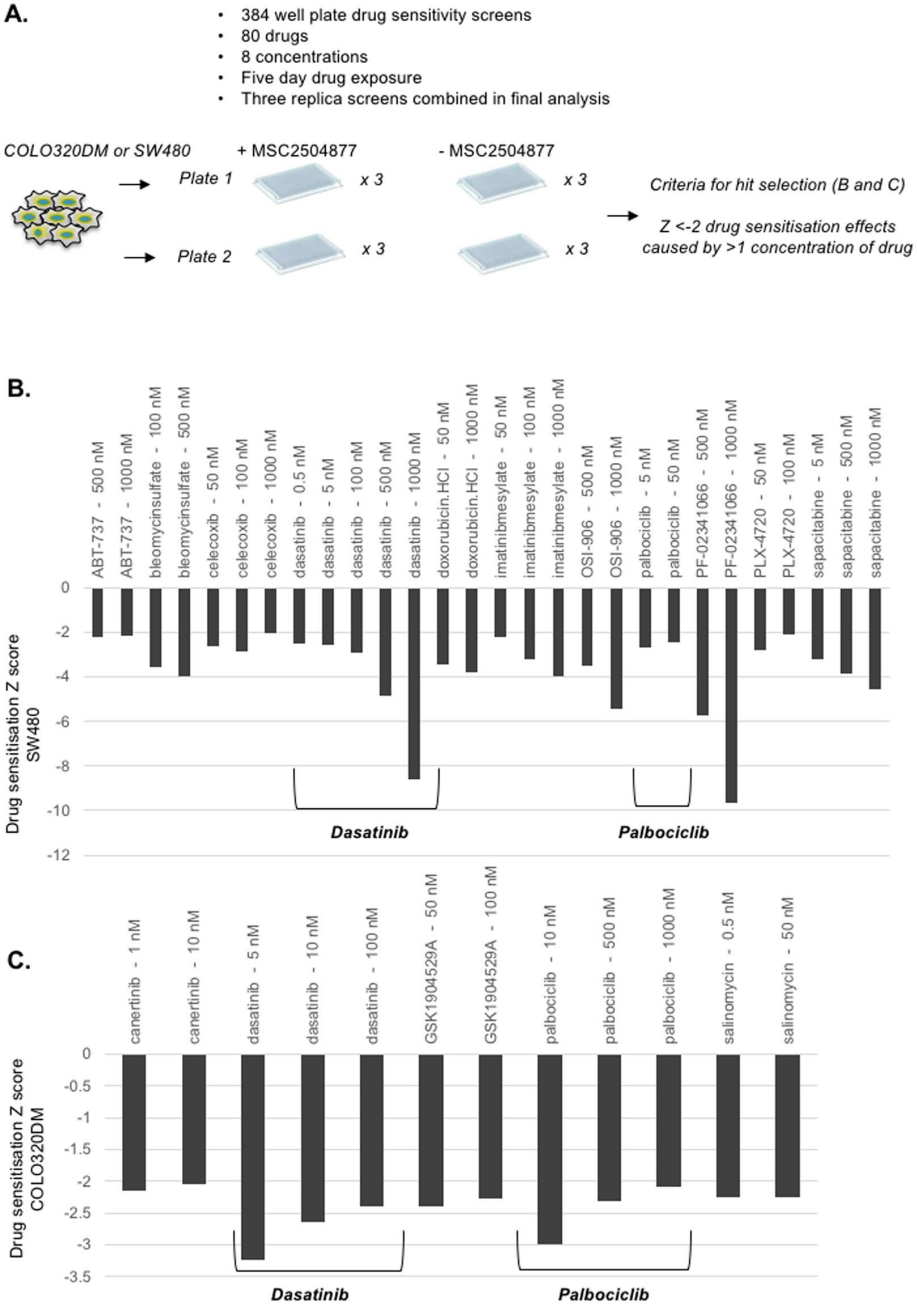
Small molecule chemosensitisation screens identify palbociclib as a candidate MSC2504877 sensitising agent. (**A**) Schematic of MSC2504877 chemosensitisation screens in colorectal tumour cells. COLO320DM and SW480 cells were exposed to 80 library drugs (at eight different concentrations, arrayed in two 384-well plates) for five continuous days, in the presence or absence of MSC2504877, at which point cell viability was estimated. Cell viability data was used to calculate drug sensitisation Z scores. Library drugs that elicited Z scores of <−2 at more than one different concentration were selected as candidate MSC2504877 chemosensitisation effects, and shown in (**B**,**C**). (**B**,**C**) Bar charts illustrating candidate MSC2504877 chemosensitisation effects in SW480 cells (**B**) and COLO320DM cells (**C**). Dasatinib and palbociclib chemosensitisation effects in both cell lines are highlighted.

For the small molecule/drug chemosensitisation screens, we plated COLO320DM cells (*APC* p.S811* homozygous and *KRAS* wild type) or SW480 cells (APC mutation p.N125K;p.F1197fs;p.S1278* heterozygous, KRAS mutation p.G12C heterozygous) in 384 well plates containing MSC2504877 or the drug vehicle (DMSO), combined with a library of 80 small molecule inhibitors that are either already used in the treatment of cancer, or are in late stage development. To maximize the likelihood of identifying chemosensitization effects, each small molecule inhibitor in the library was present at eight different concentrations. Cells were then exposed to drugs for five continuous days, at which point cell viability was estimated by the use of Cell TitreGLo reagent (Fig. 3A). Each tumour cell line was screened in triplicate, with median effects being used to estimate MSC2504877 sensitivity-causing effects. Replica screens showed a high level of reproducibility between independent replicas (r^2^ > 0.7) and a suitable dynamic range (Z’ > 0.5).

To identify candidate chemosensitization effects, we calculated Drug Sensitisation (DE) robust Z scores describing the effect of each library drug on the response to MSC2504877; we defined those library drugs that elicited DE Z < −2 effects (approximately equivalent to p < 0.05) with at least two different concentrations as being candidate sensitivity causing effects (Fig. 3B,C). Amongst these sensitization effects, we noted the presence of previously reported effects, including tankyrase inhibitor sensitization caused by an EGFR inhibitor^28^, canertinib (MSC2504877 sensitisation effect in COLO320DM) and MSC2504877 sensitization caused by the multikinase inhibitor dasatinib^19^ (MSC2504877 sensitisation effect in both SW480 and COLO320DM) giving us some confidence in the results. Our intermediate aim was to identify sensitization effects that operated in a variety of *APC* mutant colorectal tumour cell lines; by intersecting the data from both COLO320DM and SW480 screens, we identified two drugs that caused MSC2504877 sensitivity in both: the mutlikinase inhibitor, dasatinib, identified in earlier work^19^, and the CDK4/6 kinase inhibitor palbociclib (PF-332991).

**Figure 4:**
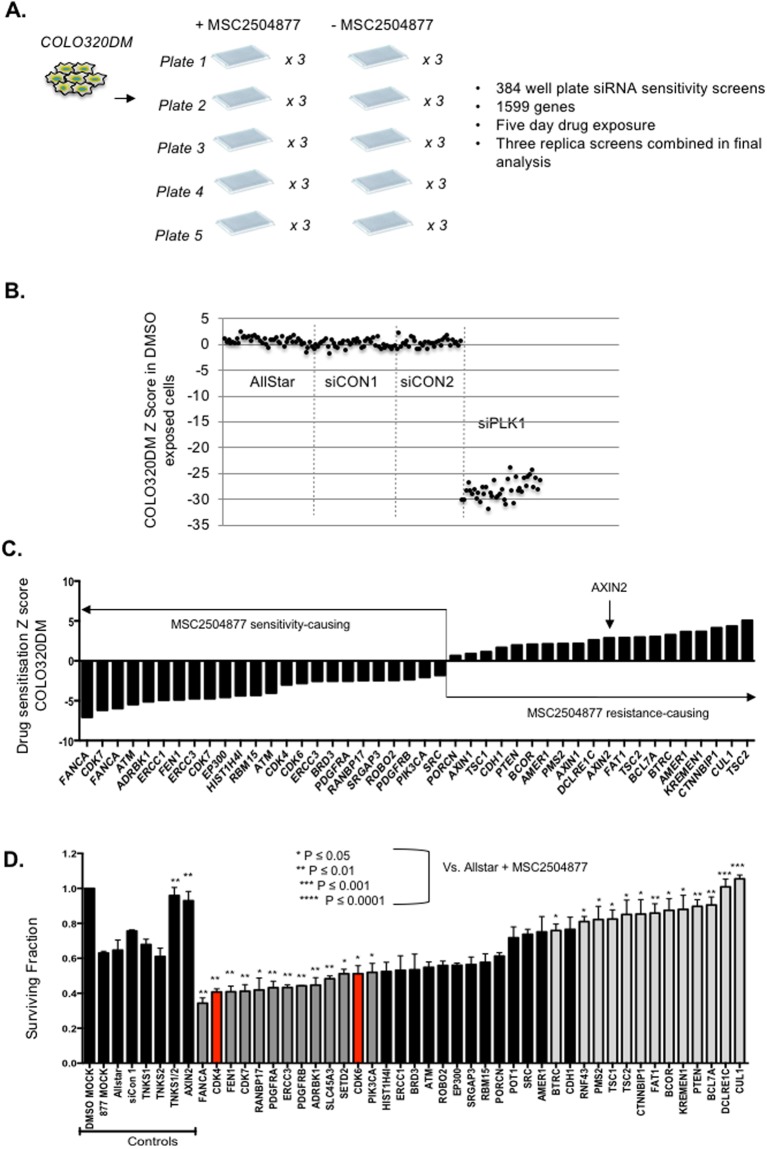
A RNA interference (RNAi) sensitisation screen identifies candidate MSC2504877 sensitivity- and resistance-causing effects. (**A**) Schematic of RNAi screen in colorectal tumour cells. COLO320DM cells were transfected with 1599 different siRNA SMARTPools (arrayed in five different 384 well-plates) and then cultured for five continuous days in the presence or absence of MSC2504877. At this point, cell viability was estimated. Cell viability data was used to calculate drug sensitisation Z scores. siRNAs that elicited Z scores of <−2 or >2 were selected as candidate MSC2504877 sensitisation/resistance-causing effects, shown in (**C**). (**B**) Scatter plot illustrating the effect of control siRNAs on cell viability in COLO320DM cells. siRNA designed to target Polo-like kinase 1 (PLK1) caused profound cell inhibition, when compared to three different non-targeting siRNAs (Allstar, siCON1 and siCON2). (**C**). Bar chart illustrating drug sensitisation Z scores of “hits” identified in the screen. (**D**) Bar chart illustrating MSC2504877 responses in post-screen validation experiments. COLO320DM cells were transfected with SMARTpool siRNA and then exposed to MSC2504877 for five days at which point cell viability was estimated. Effects of CDK4 and CDK6 siRNA SMARTpools are highlighted in red, both of which significantly enhanced MSC2504877 sensitivity, compared to control, non-targeting siRNAs (Allstar and siCON1). Dark grey columns indicate additional MSC2504877 sensitivity-causing siRNAs, whilst light grey bars indicate MSC2504877 resistance-causing effects. Validation was carried out in a 384 well-plate five day assay. Ratio of survival fraction in the MSC2504877A exposed cells compared to DMSO exposed cells is plotted. *p ≤ 0.05, **p ≤ 0.01, ***p ≤ 0.001, ****p ≤ 0.0001, Student’s t test. Error bars represent SEM from triplicate experiments.

Alongside these drug combination screens, we also carried out short interfering (si)RNA chemosensitisation screens using MSC2504877 (Fig. 4A). COLO320DM cells were reverse transfected in 384 well plates using an siRNA library designed to target 1143 genes, including a panel of protein kinases, genes included in the cancer gene census^29^ and also, genes implicated in Wnt signaling, given the role of tankyrases in this molecular pathway. Twenty-four hours after siRNA transfection, cells were either exposed to MSC2504877 or the drug vehicle for a subsequent five days, at which point cell viability was estimated by the use of Cell TitreGlo reagent (Fig. 4A). We performed three replica screens, and combined the replicas in the final analysis. Replica screens showed a high level of reproducibility between independent replicas (r^2^ > 0.7) and a suitable dynamic range (Z’ > 0.5).

To identify candidate sensitivity and resistance-causing effects, we calculated Drug Sensitisation Z scores describing the effect of siRNA on the response to MSC2504877; we defined those siRNAs that elicited Z < −2 effects (approximately equivalent to p < 0.05) as being candidate sensitivity-causing effects and, conversely defined Z > 2 effects as resistance-causing. Candidate resistance-causing or sensitivity-causing siRNAs are illustrated in Fig. 4C. We noted that one of the most profound resistance-causing effects was caused by siRNA designed to target AXIN2 (Fig. 4C), an effect that confirmed previous observations made using a first-generation small molecule tankyrase inhibitor, XAV939, in *APC* mutant DLD1 CRC cells^3^. Using the Z score thresholds of Z < −2 (sensitivity) and Z > 2 (resistance), we selected siRNAs for further analysis in validation experiments. Of the 44 siRNAs selected for validation, we found in subsequent experiments that 13 caused sensitivity to MSC2504877 (*FANCA, CDK4, FEN1, CDK7, RANBP17, ERCC3, PDGFA, PDGFRB, ADBRK1, SLC45A3, SETD2, CDK6* and *PIK3CA*) whilst 13 siRNAs caused MSC2504877 resistance (*BTRC, RNF43, PMS2, TSC1, TSC2, CTNNBIP1, FAT1, BCOR, KREMEN1, PTEN, BCL7A, DCLRE1C, CUL1* and *AXIN2*) (Fig. 4D). In addition to *AXIN2*, we found that a number of other siRNA designed to target modifiers of Wnt signaling caused MSC2504877 resistance. These included siRNAs designed to target FAT1 or CTNNBIP1, proteins which normally bind β-Catenin and impair its nuclear localization^30^, and CUL1, BTRC (β-TrCP) and AMER1 (also known as *FAM123B* or *WTX)*, proteins which normally mediate the proteosomal degradation of β-Catenin. These observations suggested that modulation of a series of additional Wnt pathway components, and specifically those that control the level of nuclear β-Catenin, could drive resistance to tankyrase inhibitors, similar to the observations made by de la Roche *et al*., who demonstrated that LEF1 and B9L, proteins that prevent Axin driven degradation of β-Catenin, reverse tankyrase inhibitor sensitivity^31^. We also noted that siRNA targeting PTEN, TSC1 or TSC2 also caused MSC2504877 resistance (Fig. 4D), implicating the PI3-kinase/mTOR signaling cascade in controlling the response to MSC2504877. In terms of sensitivity causing effects, siRNA targeting *PIK3CA*, the gene encoding the catalytic domain of the major PI3-kinase enzyme, was also confirmed (Fig. 4D).

By cross referencing the results from the small molecule chemosensitisation screens and the siRNA screen, we noted that siRNAs targeting CDK4 or CDK6 also caused sensitivity to MSC2504877 (Fig. 4C,D), corroborating the results seen with the CDK4/6 inhibitor palbociclib in the small molecule sensitisation screens (Fig. 3). We investigated this further, initially by assessing whether sensitivity to MSC2504877 caused by palbociclib was specific to the COLO320DM model or was also found in additional CRC tumour cell line models.

### Tankyrase inhibitor sensitisation by CDK4/6 inhibition is not specific to palbociclib or MSC2504877

**Figure 5:**
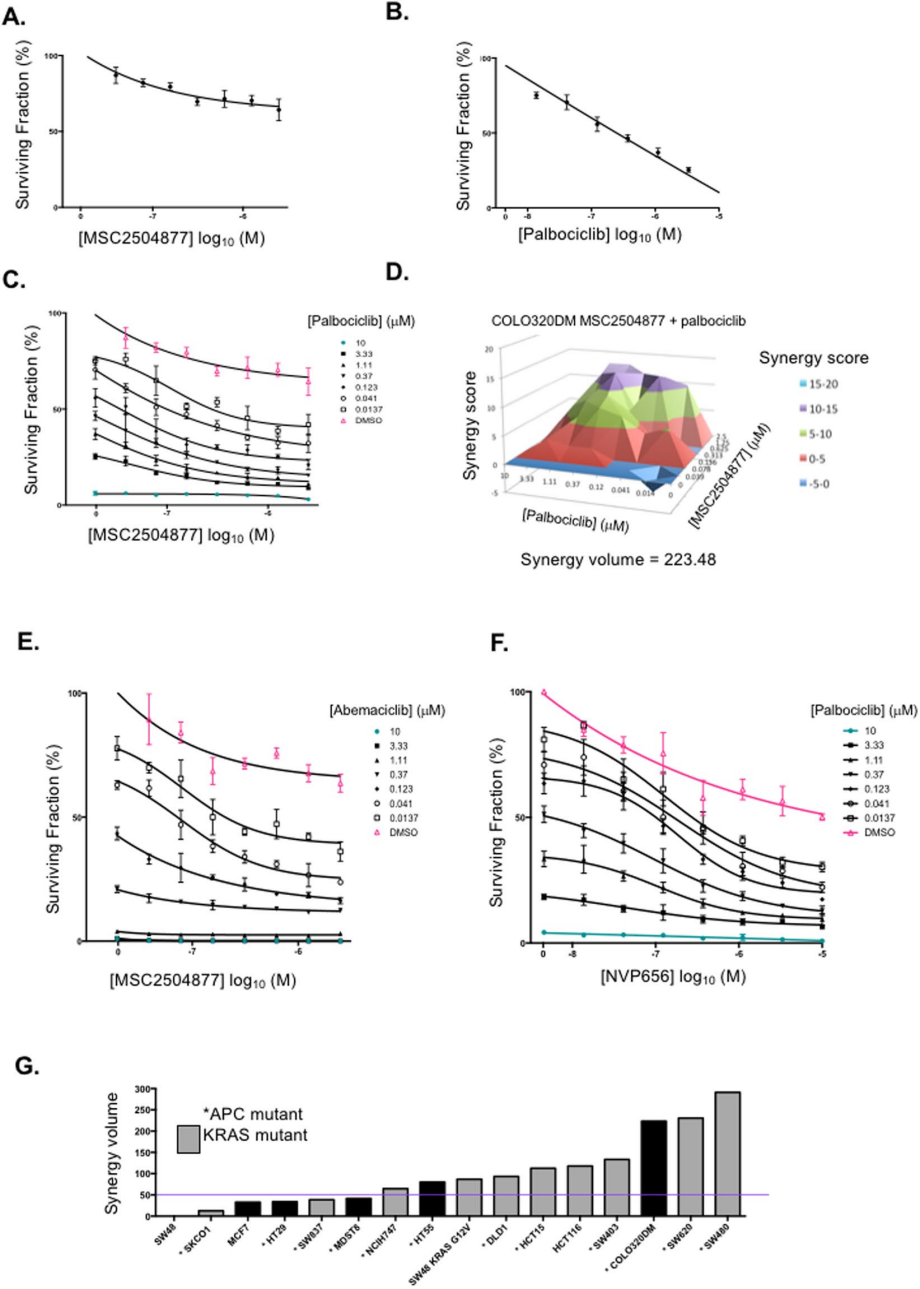
CDK4/6 inhibitors sensitise tumour cells to tankyrase inhibitors.(**A**–**C**). Dose response cell survival curve for COLO32DM cells exposed to (**A**) MSC2504877 (**B**) palbociclib, or (**C**) the combination of MSC2504877 plus palbociclib, for five days. Mean effect from three independent experiments is shown; error bars represent SEM. (**D**) Synergy volume plot indicating supra-additive effect of MSC2504877 plus palbociclib in COLO320DM cells. Synergy volumes >50 are regarded as strong synergistic effects (**E**,**F**). Dose response cell survival curve for COLO32DM cells exposed to (**E**) MSC2504877 plus abemaciclib, or (**B**) NVP656 plus palbociclib, for five days. Mean effect from three independent experiments is shown; error bars represent SEM. (**G**) Bar chart of synergy volumes from MSC2504877 plus palbociclib dose response experiments in colorectal tumour cells. Colorectal tumour cells were exposed to MSC2504877 plus palbociclib in triplicate experiments as in (**A**–**D**). Synergy volumes were calculated using MacSynergyII.

We confirmed that palbociclib caused MSC2504877 sensitivity in COLO320DM cells (Fig. 5A–D) and found that a chemically distinct CDK4/6 inhibitor, abemaciclib (LY2835219, Eli Lily), also elicited a synergstic effect with MSC2504877 (Fig. 5E). Furthermore, we found that a toolbox tankyrase inhibitor, NVP-656, also had a synergistic interaction with palbociclib (Fig. 5F). These observations suggested that the CDK4/6 inhibitor *vs*. tankyrase inhibitor synergistic effects were not specific to MSC2504877 or palbociclib. In addition, we assessed whether the MSC2504877 sensitisation effect caused by palbociclib was specific to COLO320DM cells. We found that palbociclib/ MSC2504877 synergy existed in SW480 (APC p.Q1338* homozygous, KRAS p.G12V homozygous and amplified), SW620 (APC Q1338* homozygous, KRAS p.G12V homozygous and amplified) and SW403 (APC mutation p.N125K;p.F1197fs;p.S1278* heterozygous, KRAS mutation p.G12C heterozygous) CRC tumour cell lines (Fig. 5G).

### Simultaneous exposure to MSC2504877 and palbociclib enhances G_1_/S cell cycle arrest and a senescence phenotype

CDK4 and CDK6 are cyclin dependent kinases that with their Cyclin D-family partners, work within a network of tumour suppressor proteins and oncogenes that control the progression of the cell cycle. For example, the tumour suppressor protein, CDKN2A (p16INK4a) inhibits CDK4 and CDK6, events that reduce their ability to phosphorylate and inactivate the Rb (RB1) tumour suppressor protein. This function of CDKN2A in the CDKN2A, CDK4/6, Rb signaling cascade alters the activity of a series of Rb-controlled E2F family transcription factors, which in turn suppress G_1_/S cell cycle progression. This suppression of G_1_/S cell cycle progression also often leads to cellular senescence. In the absence of *CDKN2A*, a common driver event in human cancers, the control of the G_1_/S cell cycle checkpoint is impaired. Small molecule CDK4/6 inhibitors, such as palbociclib, elicit their anti-tumour effects in part by reinstating the G_1_/S checkpoint in tumour cells that still have the ability to express Rb; this often leads to cellular senescence^32^. Palbociclib has been approved for use as part of drug combination regimens in estrogen receptor positive advanced breast cancer^33,34^. In colorectal cancers, the CDKN2A, CDK4/6, Rb signaling cascade might also control tumourigenesis driven by loss of wild type *APC* function. For example, in a mouse model of adenoma formation, *Apc* dysfunction causes an increase in Cyclin D2 and CDK4 expression and a corresponding increase in hyperphosphorylated Rb^35^. Furthermore, Cyclin D2 causes a reduction in enterocyte proliferation and crypt size in *Apc*-deficient intestinal epithelia, and CDK4/6 inhibition limits the proliferation of adenomatous cells, but not normal cells, in *Apc(Min/*+*)* mice^35^.

**Figure 6:**
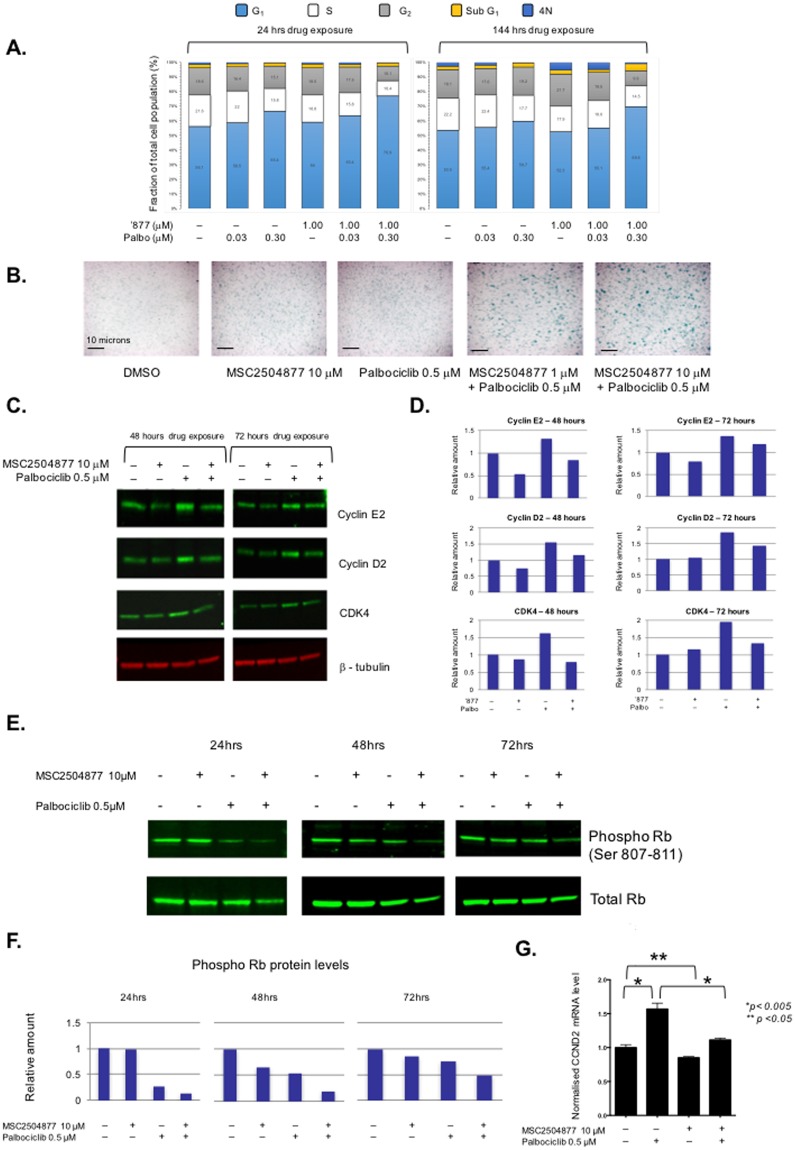
MSC2504877 + palbociclib combination elicits G_1_ cell cycle effects. (**A**) Bar chart illustrating cell cycle fractions in COLO320DM cells exposed to MSC2504877 plus palbociclib. Numbers in boxes reflect % of total cell number. (**B**) Microscopy images illustrating β-galactosidase staining in COLO320DM cells exposed to MSC2504877 plus palbociclib. Blue staining indicates β-galactosidase positive cells. Scale bar represents 10 microns. (**C**) Western blot image illustrating Cyclin D2, Cyclin E2 and CDK4 protein levels in COLO320DM cells exposed to MSC2504877. Palbociclib causes an increase in Cyclin D2 and Cyclin E2 protein levels, which is suppressed by MSC2504877. (**D**) Bar chart illustrating band densiometry measurements from (**C**). (**E**) Western blot image illustrating phospho-Rb suppression in COLO320DM cells exposed to MSC2504877 and palbociclib. Palbociclib alone causes a transient suppression in phospho-Rb levels (at 24 and 48 hours). The addition of MSC2504877 temporally extends phospho-Rb suppression. (**F**) Bar chart illustrating band densiometry measurements from (**E**). (**G**) Bar chart illustrating normalised RT-PCR measurements from COLO320DM cells exposed to MSC2504877 plus palbociclib for 24 hours. Data shown is from triplicate experiments. Error bars represent SEM.

Based on these observations, we assessed whether the combination of palbociclib and MSC2504877 caused a change in cell cycle progression. FACS profiling of COLO320DM cells demonstrated that MSC2504877 exposure alone had negligible effects on the cell cycle, whilst, palbociclib caused a modest, concentration dependent, increase in the G_1_ fraction of cells (G_1_ fraction increased from 56% to 66% in response to 0.3  μM palbociclib after 24 hours of treatment and 54% to 60% after 144 hours of treatment) (Fig. 6A). However, in contrast to the single agent effects, the combination of MSC2504877 and palbociclib caused a supra-additive increase in the G_1_ fraction (G_1_ fraction increased from 56% to 77% at 24 hours and 54% to 70% at 144 hours, Fig. 6A). We also noted that whilst single agent use of either palbociclib or MSC2504877 alone had negligible effects on the proportion of COLO320DM cells exhibiting a senescent phenotype (as estimated by β-galactosidase staining), exposure to both agents caused a clear increase in β-galactosidase positive cells (Fig. 6B). Taken together, this suggested that the synergistic effect of palbociclib when used with MSC2504877, could be explained by a supra-additive effect on the G_1_/S cell cycle checkpoint and the induction of cellular senescence, phenotypes reminiscent of a re-instated Rb-mediated signaling cascade.

To investigate the molecular features of this effect, we assessed the expression and phosphorylation status of a series of proteins involved in the CDKN2A, CDK4/6, Rb signaling cascade when cells were exposed to a palbociclib/MSC2504877 combination (Fig. 6C–E). We found that whilst exposure to palbociclib caused an increase in Cyclin D2 and Cyclin E2 levels, presumably a mechanism by which the cells override the G_1_ cell cycle blockade initiated by CDK4/6 inhibition, the addition of MSC2504877 suppressed these Cyclin D2/E2 responses (Fig. 6C,D). We also noted a time dependent effect on the levels of phosphorylated Rb (S807–811); as expected, palbociclib exposure elicited a decrease in level of phosphorylation after 24 hrs of exposure (Fig. 6E,F). However, after 72 hrs, levels of phosphorylated Rb in cells exposed to palbociclib were restored to those in cells exposed to the drug vehicle, DMSO (Fig. 6E,F). In contrast, the addition of MSC2504877 to palbociclib maintained the suppression of phosphorylated Rb levels over the 72 hour time course (Fig. 6E,F). These observations, taken together with the FACS profiling data, were consistent with the hypothesis that MSC2504877, when combined with palbociclib, reinforces G_1_ cell cycle arrest and ultimately causes cells to senesce. The CyclinD2-coding gene, *CCND2*, is a transcriptional target of canonical Wnt signalling. Using RT-PCR, we found that palbociclib exposure caused an upregulation of *CCND2* mRNA levels (Fig. 6G), commensurate with the increase in Cyclin D2 protein levels seen earlier (Fig. 6C,D); this increase in *CCND2* mRNA levels was reduced to basal levels when cells were also exposed to MSC2504877 (Fig. 6G), providing a mechanistic explanation for the combined tumour cell inhibitory effects of palbociclib and MSC2504877.

### Palbociclib plus MSC2504877 combination suppresses hyperproliferation in Apc defective cells *in vivo*

**Figure 7:**
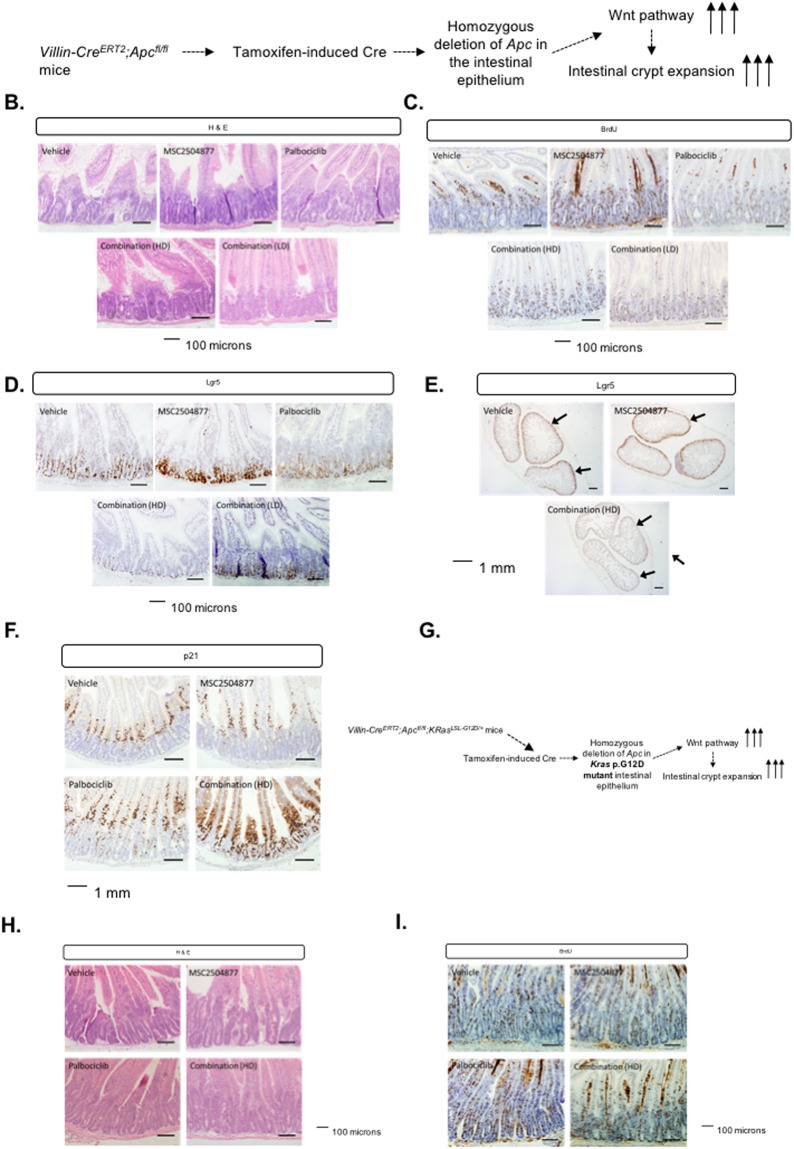
Palbociclib plus MSC2504877 combination suppresses the intestinal hyperproliferation phenotype in *Villin-Cre*^*ERT2*^*; Apc*^*fl/fl*^ mice. (**A**) Schematic of *Villin-Cre*^*ERT2*^*; Apc*^*fl/fl*^ model system. (**B**) Images of hematoxylin and eosin stained intestinal tissue from *Villin-Cre*^*ERT2*^*; Apc*^*fl/fl*^ mice. Animals received vehicle, MSC2504877, palbociclib or a combination of MSC2504877 plus palbociclib for 3 continuous days. Combination HD = high dose combination, 50 mg/kg MSC2504877 + 150 mg/kg palbociclib. Combination LD = low dose combination, 20 mg/kg MSC2504877 + 37.5 mg/kg palbociclib. (**C**) Images of intestinal tissue from *Villin-Cre*^*ERT2*^*; Apc*^*fl/fl*^ mice immunohistochemically stained for BrdU incorporation. Mice were treated as in (**B**). (**D**) Images of intestinal tissue from *Villin-Cre*^*ERT2*^*; Apc*^*fl/fl*^ mice immunohistochemically stained with an RNAscope probe directed against Lgr5 mRNA. Mice were treated as in (**B**). (**E**) Images of intestinal tissue whole mounts from *Villin-Cre*^*ERT2*^*; Apc*^*fl/fl*^ mice stained with an RNAscope probe directed against Lgr5 mRNA. Mice were treated as in (**B**). Arrows in control sample image indicates presence of Lgr5 expression; arrows in combination (TNKSi + Palbociclib) treated mice image shows absence of Lgr5 expression. (**F**) Images of intestinal tissue from *Villin-Cre*^*ERT2*^*; Apc*^*fl/fl*^ mice immunohistochemically stained for the presence of p21. Mice were treated as in (**B**). (**G**) Schematic of *Villin-Cre*^*ERT2*^*; Apc*^*fl/fl*^*; KRas*^*LSL-G12D/*+^ model system. (**H**) Images of hematoxylin and eosin stained intestinal tissue from *Villin-Cre*^*ERT2*^*; Apc*^*fl/fl*^*; KRas*^*LSL-G12D/*+^ mice. Animals received vehicle, MSC2504877, palbociclib or a combination of MSC2504877 plus palbociclib. Combination HD = high dose combination, 50 mg/kg MSC2504877 + 150 mg/kg palbociclib. Combination LD = low dose combination, 20 mg/kg MSC2504877 + 37.5 mg/kg palbociclib. (**I**) Images of intestinal tissue from *Villin-Cre*^*ERT2*^*; Apc*^*fl/fl*^*; KRas*^*LSL-G12D/*+^ mice immunohistochemically stained for BrdU incorporation. Mice were treated as in (**H**).

To assess whether these effects operated in an *in vivo* setting, we assessed the impact of a MSC2504877 + palbociclib combination in genetically engineered mice carrying *Villin-Cre*^*ERT2*^ and homozygous for the *Apc*^*fl*^ allele; tamoxifen-induced, Cre-mediated recombination in this mouse model drives homozygous deletion of *Apc* in the entire intestinal epithelium, aberrant activation of the Wnt pathway and as consequence, dramatic intestinal crypt expansion^36^ (Fig. 7A). In the first instance, we dosed *Villin-Cre*^*ERT2*^*; Apc*^*fl/fl*^ mice with either single agent MSC2504877 (50 mg/kg), single agent palbociclib (150 mg/kg), or a MSC2504877 (50 mg/kg) + palbociclib (150 mg/kg) combination. Whilst neither single agent drug administration suppressed intestinal crypt expansion caused by *Apc* deletion, the combination of MSC2504877 with palbociclib had a dramatic suppressive effect, as seen both by the gross histology of tissue sections isolated from mice (Fig. 7B), and also a profound reduction in bromodeoxyuridine (BrdU) incorporation in intestinal crypts (Fig. 7C). These effects were apparent using a 50 mg/kg MSC2504877 + 150 mg/kg palbociclib combination (“high dose combination”, (HD)), as well at lower concentrations of MSC2504877 + palbociclib (20 mg/kg + 37.5 mg/kg, respectively, “low dose combination” (LD) Fig. 7B,C). Combined exposure to MSC2504877 + palbociclib also suppressed the expression of the archetypal Wnt target gene and stem cell marker *Lgr5* (Fig. 7D,E) and combination drug treatment caused a profound increase in nuclear p21 (Fig. 7F), consistent with an effect on mechanisms of G_1_/S cell cycle control.

In many human tumours, including colorectal cancers, loss of function *APC* mutations co-occur with mutation in *KRAS*. We also exploited genetically engineered mice to assess whether the presence of a *Kras* mutation might modulate the effects of a tankyrase inhibitor/CDK4/6 inhibitor combination. To do this, we assessed the crypt hyper-proliferative phenotype in *Villin-Cre*^*ERT2*^*; Apc*^*fl/fl*^*; KRas*^*LSL-G12D/*+^ mice^37^ (Fig. 7G). By examination of the gross histology of tissue sections (Fig. 7H), and BrdU incorporation (Fig. 7I), we found that the presence of the *KRas*^*G12D*^ mutation reversed the inhibitory effects of the MSC2504877 + palbociclib combination.

## Discussion

Here we describe the characterisation of a novel tankyrase inhibitor, MSC2504877. Along with biochemical and cellular assays carried out to assess its potency and selectivity, we demonstrate that combination with CDK4/6 inhibitors has a supra-additive effect on tumour cells. Dissection of this effect suggests that this combinatorial effect might be mediated by the ability of MSC2504877 to suppress the upregulation of Cyclin D2 levels. This appears to be a distinct mechanism to those proposed for other tankyrase inhibitor combinations. For example, the supra-additive effect of a tankyrase inhibitor combined with a MEK inhibitor is proposed to be mediated via the ability of tankyrase inhibition to suppress a FGFR2-mediated feedback loop that is otherwise activated by MEK inhibition^19^. Tankyrase inhibitors have been also shown to sensitise lung cancer cells to EGFR inhibition through a mechanism that involves stabilisation of angiomotins and inhibition of YAP signalling^5^. It thus seems reasonable to propose that there might be multiple, distinct, routes by which tankyrase inhibition alter the homeostatic response to other drugs.

One challenge associated with the development of tankyrase inhibitors as a treatment for cancer has been the intestinal toxicity observed in pre-clinical animal models. Wnt/β catenin signalling maintains homeostasis in the intestinal epithelium; inhibition of Wnt/β catenin signalling results in terminal differentiation of stem cells and loss of crypt structure^38,39^. It seems possible that the suppression of Wnt/β catenin signalling via tankyrase inhibition in intestinal stem cells is an explanation, at least in part, for the intestinal toxicity seen with tankyrase inhibitors. This in turn, might challenge the concept of therapeutically targeting canonical Wnt signalling as a therapeutic approach. However, preliminary data from a phase 1 clinical trial of WNT974, an inhibitor of porcupine (a membrane-bound O-acyltransferase enzyme required for Wnt ligand secretion), reports a manageable safety profile as well as suppression of a key Wnt/β catenin target gene, *AXIN2*^40^. Whether CDK4,6 inhibitors might also have combinatorial effects with Porcupine inhibitors or other drug targets in Wnt/β catenin signalling cascade remains to be seen.

In summary, MSC2504877 is a novel, drug-like, tankyrase inhibitor that inhibits *APC* mutant colorectal tumour cells. Parallel siRNA and drug sensitivity screens show that the clinical CDK4/6 inhibitor palbociclib, causes enhanced sensitivity to MSC2504877. This tankyrase inhibitor - CDK4/6 inhibitor combinatorial effect is not limited to palbociclib and MSC2504877 and is elicited with other CDK4/6 inhibitors and other tankyrase inhibitors, namely NVP-656. Nevertheless, it is possible that these effects are private to the tankyrase inhibitors/CDK4/6 inhibitors tested. The addition of MSC2504877 to palbociclib enhances G_1_ cell cycle arrest and cellular senescence. MSC2504877 exposure suppresses the upregulation of Cyclin D2 and Cyclin E2 caused by palbociclib and enhances the suppression of phopho-Rb. Combinatorial effects of MSC2504877 and palbociclib are also seen in suppressing the cellular hyperproliferative phenotype seen in Apc defective intestinal stem cells *in vivo*.

## Electronic supplementary material

Supplementary Information
