# Supplementary material for: A novel tankyrase inhibitor, MSC2504877, enhances the effects of clinical CDK4/6 inhibitors
Source: Sci Rep. 2019 Jan 17;9:201. doi: 10.1038/s41598-018-36447-4 (PMC6336890; doi:10.1038/s41598-018-36447-4)

A novel tankyrase inhibitor, MSC2504877, enhances the effects of clinical CDK4/6 inhibitors

Malini Menon[1], Richard Elliott[1], Leandra Bowers[1], Nicolae Balan[1], Rumana Rafiq[1], Sara Costa-Cabral[1], Felix Munkonge[1], Ines Trinidade[1], Roderick Porter[2], Andrew D. Campbell[3], Emma R. Johnson[3], Christina Esdar[4], Hans-Peter Buchstaller[4], Birgitta Leuthner[4], Felix Rohdich[4], Richard Schneider[4], Owen Sansom[3], Dirk Wienke[4]*, Alan Ashworth[1,5]* and Christopher J. Lord[1]*

[1]CRUK Gene Function Laboratory and Breast Cancer Now Toby Robins Breast Cancer Research Centre, The Institute of Cancer Research, London, SW3 6JB, UK

[2]The Wellcome Trust, Euston Road, London, NW1 2BE, UK

[3]CRUK Beatson Institute, Switchback Rd, Bearsden, Glasgow, G61 1BD, UK

[4]Merck KGaA, Biopharma Research & Development, Frankfurter Str. 250, 64293 Darmstadt, Germany

[5]Current Address: UCSF Helen Diller Family Comprehensive Cancer Centre, San Francisco, USA 94158

*To whom correspondence should be addressed:

Dirk.Wienke@merckgroup.com

Alan.Ashworth@ucsf.edu

Chris.Lord@icr.ac.uk

SUPPLEMENTARY INFORMATION

Supplementary Figure 1: Dose response curve showing activity of MSC2504877A and other previously published Tankyrase inhibitors on TCF dependent transcription in the colorectal cancer cell line SW480. The cell line stably expresses luciferase driven by a TCF-dependent promoter.

Supplementary Figure 2. mRNA levels of RUNX2 and AXIN2 are suppressed by exposure to MSC2504877. COLO320DM cells were exposed to 1 μM MSC2504877 for 1 hour, at which point mRNA was extracted from cells. qPCR was carried out using ThermoFisher TaqMan probes according to the manufacturer's instructions. mRNA levels compared to those of a housekeeping gene, GAPDH, are shown. Error bars represent standard error of the mean from three replica experiments. In each case the target gene is suppressed $p<0.05$ (Student's t test) in MSC2504877-exposed cells, compared to cells exposed to the drug vehicle, DMSO.

Supplementary Figure 1.

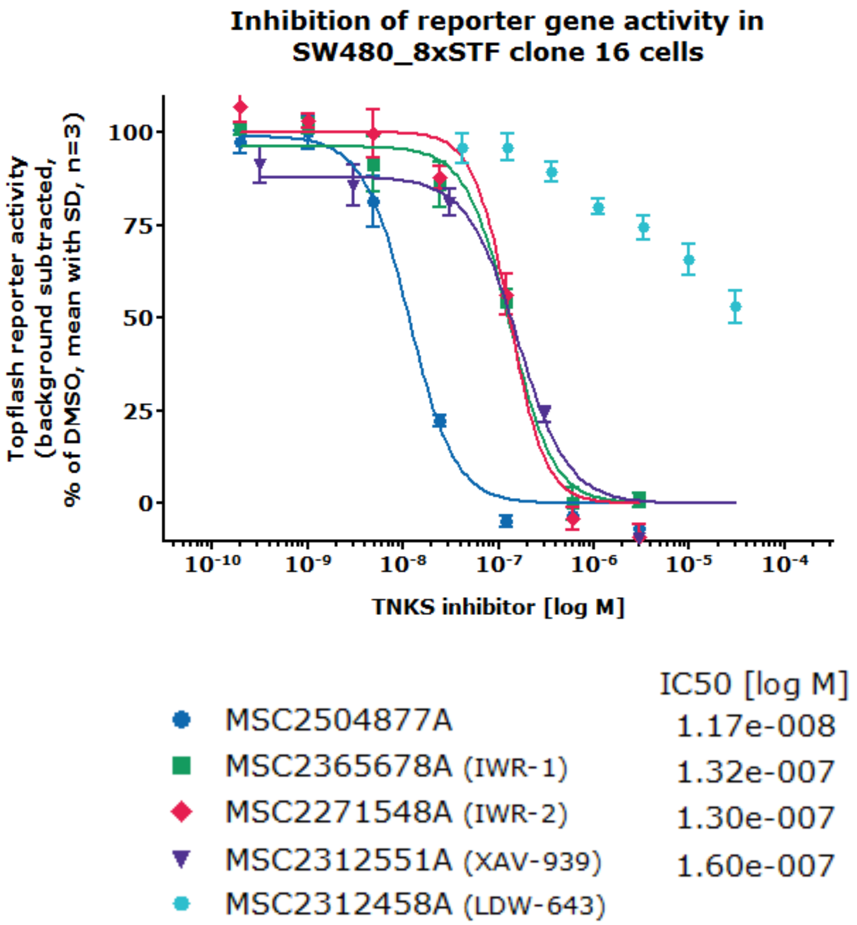

Supplementary Figure 2.

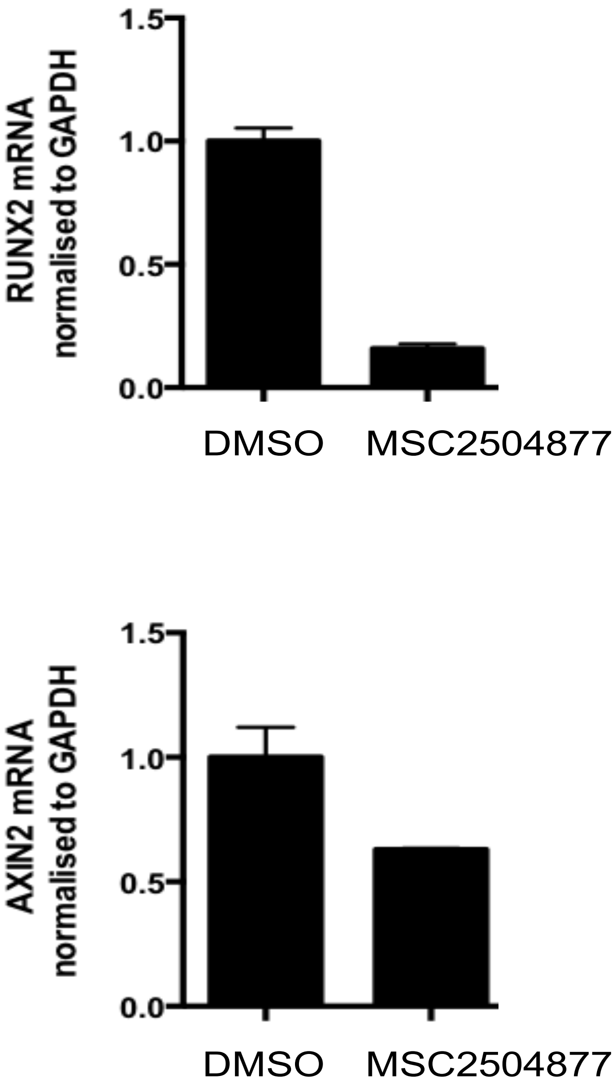

Supplement: Supplementary file 1 — Supplementary Information [file 41598_2018_36447_MOESM1_ESM.pdf]
